# Role of glucocorticoids in neutrophil and endothelial adhesion molecule expression and function

**DOI:** 10.1155/S0962935192000176

**Published:** 1992

**Authors:** Kevin D. Forsyth, Vivienne Talbot

**Affiliations:** 1 Department of Immunology Princess Margaret Hospital for Children Box D184 GPO Perth 6001 Australia

## Abstract

Glucocorticoids are very effective inhibitors of both the acute and chronic inflammatory response. In this study the hypothesis that glucocorticoids inhibit an early component of the inflammatory response, neutrophil adhesion to endothelium, by down-regulation of adhesion molecules on neutrophils or endothelium was examined. No effect of dexamethasone on neutrophil adhesion to endothelium or of antigen expression by neutrophils or endothelium was found. The mechanism of action of glucocorticoids in the inflammatory response is probably not mediated by alterations in adhesion molecules.


Mediators of Inflammation, 1, 101–106 (1992)

### Role of glucocorticoids in neutrophil and endothelial adhesion molecule expression and function

**Kevin D. Forsyth[CA] MB ChB MD PhD FRACP FRCPA and Vivienne Talbot, NZCS, ANZMILT**

Department of Immunology,
Princess Margaret Hospital for Children,
Box D184, GPO Perth 6001,
Western Australia

[CA] Corresponding Author

**GLUCOCORTICOIDS are very effective inhibitors of both the acute and chronic inflammatory response. In this study the hypothesis that glucocorticoids inhibit an early component of the inflammatory response, neutrophil adhesion to endothelium, by down-regulation of adhesion molecules on neutrophils or endothelium was examined. No effect of dexamethasone on neutrophil adhesion to endothelium or of antigen expression by neutrophils or endothelium was found. The mechanism of action of glucocorticoids in the inflammatory response is probably not mediated by alterations in adhesion molecules.**

**Key words:** Dexamethasone, Endothelial antigen expression, Glucocorticoids, Neutrophil adhesion

### Introduction

For many years glucocorticoids have been recognized as having important anti-inflammatory properties. A report from the 1940s testified to the efficacy of glucocorticoids in patients with rheumatoid arthritis.[1] These drugs are used clinically in many of the acute and chronic inflammatory conditions and in the vasculitidies.[2]

Of particular recent interest is the use of dexamethasone in the treatment of acute inflammatory conditions, where host-cell directed tissue injury, particularly neutrophil mediated, is thought to contribute to disease morbidity and mortality. One such condition in the paediatric population is bacterial meningitis,[3,4] with the majority of North American pediatric infectious disease program directors utilizing glucocorticoid therapy in the treatment of acute bacterial sepsis and meningitis.[5] Dexamethasone is also recommended for use in preterm infants at risk of developing bronchopulmonary dysplasia.[6] However, in another condition where neutrophils are considered to induce pulmonary pathology, Adult Respiratory Distress Syndrome (ARDS), glucocorticoids have no discernible benefit.[7] The reasons for this lack of effect are unclear, and illustrate that in spite of such widespread acceptance of glucocorticoid therapy, understanding the mode of action of these anti-inflammatory agents has been slow.

Glucocorticoids are thought to modulate neutrophil–endothelial cell interactions, a key area in the acute inflammatory response. It has been observed that following *in vivo* administration of glucocorticoids, there is a moderate neutrophilia within 4–6 h.[8] Mechanisms of this are unclear, but are thought to include delayed bone marrow release and intravascular retention.[8] Continued administration of glucocorticoids induces a progressive rise in peripheral blood neutrophil counts until day 7, followed by a steady fall to baseline levels.[9]

As glucocorticoids inhibit the inflammatory response, and neutrophil adhesion to endothelium is an early component of the inflammatory response, in this paper the hypothesis that glucocorticoids inhibit neutrophil adhesion to endothelium (thereby inducing a neutrophilia in a manner analogous to that found in patients with leukocyte adhesion deficiency) by down-regulating adhesion molecules on neutrophils or endothelium is examined.

**Abbreviations:** LPS, lipopolysaccharide; NBCS, newborn calf serum; FMLP, Phe-Met-Leu-Phe; MHC, major histocompatibility complex; ICAM-1, intercellular adhesion molecule 1; ELAM-1, endothelial leukocyte adhesion molecule 1; IL-1, interleukin 1; IL-2, interleukin 2; TNF, tumour necrosis factor; $LTB_4$, leukotriene $B_4$.

### Methods

*Endothelial culture:* Endothelial cells were obtained from human umbilical cords collected daily from a

© 1992 Rapid Communications of Oxford Ltd maternity hospital. From the time of delivery until endothelium cell harvest, the cords were stored in sterile RPMI 1640 (Gibco) containing 80 U/ml gentamicin (David Bull Laboratories). Endothelial cells were harvested by digestion of the veins for 15 min at 37°C with collagenase type II (Sigma, C 6885, St Louis) 0.1% reconstituted in Dulbecco's MEM without pyruvate with 4 500 mg/l glucose (Gibco 043-5630, Paisley, Scotland). The cells were cultured in endothelial cell growth medium consisting of ISCOVES Cell Culture Medium (Gibco), supplemented with 10% foetal calf serum and 10% newborn calf serum (Flow), gentamicin 80 U/ml (David Bull Laboratories), 0.02% amphotericin B (Squibb), and 2 mM glutamine. Cells were cultured at 37°C in a 5% $CO_2$ atmosphere in 25 $cm^2$ tissue culture flasks (Linbro). By inversion microscopy the extent of confluence of the cells was assessed daily. When confluent they were passaged by exposing the endothelial cells to 0.05% trypsin and 0.02% EDTA in modified Puck's saline A (Gibco 043-5300) for 3 min at 37°C, followed by washing and re-seeding into new culture flasks. Endothelial cells were used at the second to third passage. Before use, endothelial cells were transferred to Linbro 96-well tissue culture grade microtitre plates for assessment of adhesion, or to glass coverslips for endothelial antigen expression analysis. Prior to assay the endothelial cells were confirmed visually to be confluent by inversion microscopy; this generally occurred 3–5 days after seeding.

*Endothelial stimulants:* Endothelium was stimulated with LPS (lipopolysaccharide, Sigma, St Louis) at 100 ng/ml for 20 h prior to analysis.

*Endothelial immunophenotyping:* Immunostaining of cultured endothelium was performed on either unstimulated or LPS stimulated endothelium. The biotin–streptavidin horseradish peroxidase technique was used to identify surface antigens on endothelium—in our hands this technique is more sensitive and specific than either ELISA or flow cytometric estimation of endothelial antigen expression. Coverslips which were confluent with endothelium were mounted, endothelial surface uppermost, on to glass slides with Loctite glass glue 640. Slides were fixed in cold 100% ethanol for 10 min, and then rehydrated in decreasing concentrations of ethanol. To rehydrated endothelium was added a 1:50 dilution of goat serum to block Fc receptors. This was incubated at room temperature for 30 min. Excess goat serum was flicked off the coverslip, and, without washing, 100 $\mu l$ of each antibody to be tested at predetermined optimal dilution was pipetted onto each coverslip and incubated at room temperature for 30 min in a moist chamber. The slides were washed three times in Dulbecco's phosphate buffered saline (DPBS) with 1% NBCS followed by 100 $\mu l$ of anti-mouse IgG Biotin (Amersham), reincubated for a further 30 min and washed in DPBS. To each coverslip 100 $\mu l$ of streptavidin horseradish peroxidase (Amersham) was added and the reincubation and washing procedure repeated.

The slides were then flooded for approximately 10 min with a 0.12% solution of diaminobenzidine tetrahydrochloride (Sigma) containing 5 $\mu l$ of hydrogen peroxide (Gurr) added just prior to use, washed three times in DPBS with NBCS, and counterstained for 30 s with Mayer's haematoxylin.

Slides were dehydrated in increasing concentrations of ethanol and then washed in xylene. Slides were mounted with coverslips using DePex (Gurr) mounting medium and examined microscopically.

The pattern and intensity of staining were assessed by two observers. A subjective scoring system was used to objectify the particular pattern seen. This scoring was as follows:

| | |
|---|---|
| Negative | No immunostaining present. |
| 1+ | Light immunostaining. |
| 2+ | Moderate immunostaining involving >90% of cells. |
| 3+ | Heavy immunostaining involving >95% of cells. |

*Antibodies:* Endothelium was immunostained with the antibodies listed in Table 1. Neutrophils were immunostained for flow cytometric analysis with the antibodies listed in Table 2. Negative control antibodies used were Coulter Clone control (Coulter Immunology, Florida) purified mouse IgG (all isotypes).

*Preparation of neutrophils:* All care was taken throughout the preparation stages to avoid neutrophil activation. Venous blood from normal adult donors (laboratory or hospital staff) was

**Table 1.** Antibodies directed against endothelial antigens

| CD/Group | Antibody | Isotype | Reference/ Company |
|---|---|---|---|
| MHC Class I | W6/32 | G2a | ref. 10 |
| MHC Class II | HLA-DR | G2a | DAKO |
| CD29 | 4B4 | G1 | Coulter |
| CD54 (ICAM-1) | 6.5 B5 | G | ref. 11 |
| ELAM-1 | 1.2 B6 | G | ref. 11 |
| Cell fibronectin | FN3 | G | ref. 12 |
| α-1 antichymotrypsin | DF57 | G | D. Flavell, Southampton |

Anti-CD29 (4B4) recognizes the common $\beta$ chain of the $\beta 1$ integrin family. ICAM-1 (CD54, antibody 6.5 B5) and ELAM-1 (antibody 1.2 B6) are adhesion molecules. Cellular fibronectin (FN3 antibody) is an extracellular matrix adhesive protein and a ligand for many members of the integrin family. Antibody DF57 recognizes the anti-protease α-1 antichymotrypsin, present on endothelial cells.

**Table 2.** Antibodies directed against neutrophil antigens

| CD/Group | Antibody | Isotype | Ref./Company |
|---|---|---|---|
| MHC Class I | W6/32 | G2a | ref. 10 |
| MHC Class II | HLA-DR | G2a | DAKO |
| CD18 | MHM23 | G1 | DAKO |
| CD11b | Leu 15 | G2a | Becton-Dickenson |

CD18 and CD11b are members of the $\beta 2$ leukocyte integrin family, and play an important role in neutrophil adhesion to endothelium.

collected into preservative-free heparin or EDTA, diluted with an equal volume of DPBS, and underlayered with Ficoll-Faque (Pharmacia, Uppsala, Sweden). After centrifugation at $400 \times g$ for 20 min, the pellet containing red cells and neutrophils was isolated. Most of the red cells were removed by sedimentation with 3% sterile dextran (Fisons, Loughborough) for 30 min. The remaining red cells were removed by a hypotonic lysis step, consisting of a 10 s exposure to double distilled water, followed by reconstitution in double strength DPBS with 1% foetal calf serum (FCS). Cells were recentrifuged at $200 \times g$ for 5 min, counted in a haemocytometer, and reconstituted to $1\text{–}2 \times 10^6$ cells/ml in colourless (without phenol red) RPMI 1640 (Gibco, Paisley, Scotland) containing 1% FCS. Neutrophils were used within 1 h. Lack of the phenol red dye in this RPMI meant the pH of the solution had to be checked each time it was used. The pH was maintained between 7.3 and 7.5. As the optical density of the neutrophil suspension was used to quantitate the cells (*vide infra*), a colourless buffer was preferred. Neutrophils prepared in this way were shown morphologically to be about 96% pure. There were approximately 4% contaminating eosinophils in this preparation, as judged by a cytospin preparation using a modified May Grunwald stain (consisting of Geimsa's stain 6 mg, May Grunwald stain 5 mg, methanol 4 ml, pH 6.8 buffered water 9.5 ml and 30% Brij 35, 5 $\mu$l) and observed microscopically. Siliconized plasticware was used at all stages of cell preparation.

*Neutrophil adhesion to endothelium:* This was performed utilizing the alkaline phosphatase method, described in detail previously.[13] The principle of this assay is that neutrophils contain endogenous alkaline phosphatase. If an alkaline phosphatase substrate which changes colour by the action of alkaline phosphatase is added, the resultant optical density gives an estimation of the number of alkaline phosphatase-containing neutrophils present. If the optical density of the starting population of neutrophils is compared with that of the adherent cells, the percentage adherent can be estimated.

Briefly, 100 $\mu$l of neutrophil suspension ($1.5 \times 10^6$ cells/ml) was added to confluent endothelium grown on 96-well microtitre plates (Linbro). These were incubated for 30 min, washed to remove non-adherent neutrophils, and 100 $\mu$l of buffer (colourless RPMI +1% FCS) replaced into the wells. Hence these wells contained only adherent neutrophils. To quantitate these adherent cells, a standard curve of neutrophil numbers was constructed by adding to unused wells 100 $\mu$l of the original neutrophil suspension, and in duplicate making doubling dilutions of the original neutrophil suspension. Neutrophil numbers were then counted using an alkaline phosphatase EIA (enzyme immunoassay).

*Alkaline phosphatase EIA:* One hundred microlitres of 1% p-nitrophenyl phosphate disodium (Sigma, St Louis) in diethanolamine buffer (1 M, pH 9.8) was added to each well, and incubated at 37°C for 3 to 4 h. The optical density of each well was measured at 405 nm in a microplate scanner (Biotek Instruments). Adherent cell numbers were estimated by interpolation from the standard curve. Dexamethasone pre-incubation of neutrophils did not alter alkaline phosphatase levels.

*Flow cytometric analysis of neutrophil receptors:* The fluorescent intensity of neutrophils (either unstimulated or stimulated with $10^{-7}$M FMLP) incubated with the leukocyte integrin antibodies, either pretreated with dexamethasone or on their own, was assessed by reference to the mode fluorescent intensity as provided by Becton Dickinson's Consort 30 Program linked to a FACScan™.

*Role of dexamethasone:* To assess the role of glucocorticoids on neutrophil or endothelial antigen expression and adhesion, dexamethasone (David Bull Laboratories) at final concentrations of 0.01, 0.1 and 1 $\mu$M was tested. Differing time periods of coincubation of dexamethasone with endothelium were assessed, ranging from 30 min to 24 h. In the majority of experiments assessing the effect of dexamethasone, endothelium was pretreated for 6 h. To assess the effect of dexamethasone on neutrophils, these two substances were incubated together for 2 h.

*Statistics:* The assessment of endothelial immunophenotype was performed on six separate occasions. Adhesion assays, done in quadruplicate, were performed on five separate occasions. The flow cytometry experiments were performed on four separate occasions. Error bars refer to $\pm 1$ SD from the mean. Differences between means were compared by a *t*-test; significant if $p < 0.05$.

### Results

*Effect of dexamethasone on immunophenotype:*

*Effect of dexamethasone on endothelial antigen expression.* Coincubation of dexamethasone with cultured unstimulated endothelium at doses of 0.01 μM to 1.0 μM had no effect on the endothelial expression of antigens tested (MHC Class I, Class II, CD29, ICAM-1, ELAM-1, cellular fibronectin, α-1 antichymotrypsin). Endothelium stimulated with LPS becomes activated, altering its antigen expression (Table 3). Pretreatment of endothelium with dexamethasone for 6 h prior to LPS stimulation was unable to abrogate the activation changes induced by LPS. After 20 h of LPS stimulation, there is increased endothelial expression of adhesion molecules ICAM-1 and ELAM-1, with a lesser increase in MHC Class II expression. Endothelium pretreated with dexamethasone for 6 h prior to the addition of LPS for a further 20 h showed no abrogation in adhesion molecule expression. In particular dexamethasone was unable to inhibit ELAM-1 or ICAM-1 induction by LPS.

*Effect of dexamethasone on neutrophil integrin expression.* Neutrophils stimulated by FMLP increase expression of the leukocyte integrins as assessed by the mode fluorescent intensity on flow cytometry. Dexamethasone pretreatment of neutrophils stimulated by FMLP was unable to abrogate such changes (Table 4).

*Effect of dexamethasone on neutrophil adhesion to endothelium:*

*Effect of dexamethasone on neutrophil adherence to endothelium.* Dexamethasone preincubated with resting endothelium for up to 24 h prior to the neutrophil adhesion assay had no effect on baseline neutrophil adhesion to endothelium.

*Effect of dexamethasone on neutrophil adhesion to LPS stimulated endothelium.* Activation of endothelium by treatment with LPS increases neutrophil adherence to endothelium. Prior co-culture of endothelium with dexamethasone was unable to abrogate this increase (Fig. 1).

*Effect of dexamethasone on FMLP stimulated neutrophil adhesion to LPS stimulated endothelium.* In acute inflammatory states it is likely that both the endothelium and circulating neutrophils are activated. In an attempt to mimic such a state, the endothelium was incubated with LPS, with the neutrophils added in the adhesion assay stimulated by $10^{-7}$M FMLP. Dexamethasone pre-treatment of the activated endothelium was unable to inhibit the augmented adherence of neutrophils stimulated with FMLP to endothelium stimulated with LPS (Fig. 2).

**Table 3.** Endothelial antigen expression

| Antibody | CD | Expression | | |
|---|---|---|---|---|
| | | Unstim | Post LPS 20 h | Post dexamethasone 6 h plus 20 h |
| W6/32 | Class I | 3+ | 3+ | 3+ |
| HLA-DR | Class II | 0–1+ | 2+ | 2+ |
| 4B4 | CD29 | 3+ | 3+ | 3+ |
| 6.5 B5 | CD54 | 1+ | 2+ | 2+ |
| 1.2 B6 | ELAM-1 | 0 | 2+ | 2+ |
| FN3 | — | 3+ | 3+ | 3+ |
| DF57 | — | 3+ | 3+ | 3+ |

Expression on endothelium by immunoperoxidase staining, either unstimulated or after LPS stimulation, of the above antigens. Expression is given as 0–3+, depending on staining intensity. Dexamethasone preincubation of endothelium is unable to inhibit the LPS-induced increases in Class II, ICAM-1 and ELAM-1 expression.

**Table 4.** Neutrophil integrin expression and effect of dexamethasone

| Antigen | Mode fluorescence (resting) | Mode fluorescence (post FMLP) | Mode fluorescence (post dex + FMLP) |
|---|---|---|---|
| Class I | 336 ± 14 | 346 ± 15 | 307 ± 24 |
| Class II | 8 ± 1 | 9 ± 1 | 9 ± 1 |
| CD18 | 702 ± 35 | 746 ± 12 | 741 ± 19 |
| CD11b | 667 ± 22 | 791 ± 37 | 793 ± 42 |

Mode fluorescent intensity of neutrophils for the antigens listed either resting, after FMLP stimulation, or after dexamethasone incubation followed by FMLP stimulation. The FMLP increases mode fluorescent intensity of neutrophils for CD18 and CD11b-dexamethasone is unable to abrogate this increase.

### Discussion

In this study dexamethasone was found to have no discernible effect on both stimulated or unstimulated neutrophil adhesion to stimulated or

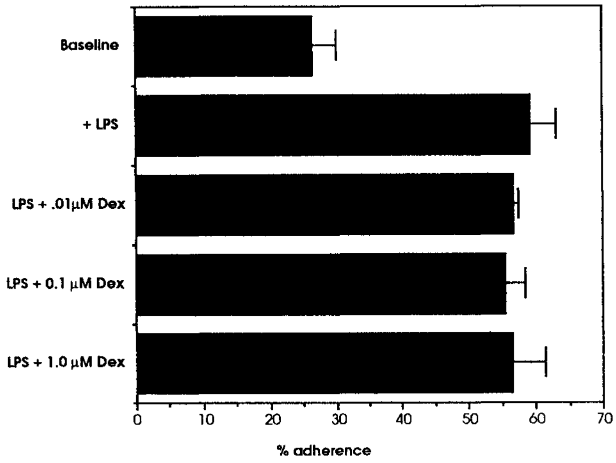

FIG. 1. Dexamethasone effect on neutrophil adherence to LPS-stimulated endothelium. Neutrophil adherence, either baseline, or to endothelium stimulated for 20 h with LPS. To dexamethasone pretreated endothelium was added LPS for 20 h, and neutrophil adherence assessed. LPS increases neutrophil binding to endothelium. Dexamethasone at concentrations of 0.01 to 1 μM is unable to inhibit this augmented adherence.

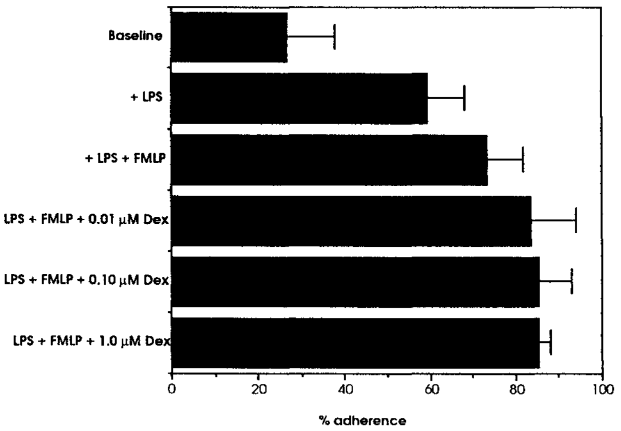

FIG. 2. Dexamethasone effect on activated neutrophil adherence to activated endothelium. LPS stimulated endothelium and FMLP stimulated neutrophils induces a hyperadherent state. Dexamethasone pretreatment of endothelium does not abrogate this adherence.

unstimulated endothelium, or on the expression of neutrophil or endothelial adhesion molecules.

The precise mechanism of action of glucocorticoids is unclear. Glucocorticoids are thought to exert their effect by inducing synthesis of lipocortins.[14] In turn, lipocortins are considered to inhibit phospholipase $A_2$ production of arachidonic acid and the eicosanoids,[15] specifically by blocking mRNA synthesis and therefore expression of group II phospholipase $A_2$.[16] Dexamethasone has been reported to reduce levels of IL-1 and TNF in the CSF of experimentally infected animals,[17] and it is thought that glucocorticoids alter gene expression of a number of key proteins in the inflammatory response, for example decreasing IL-1 production.[18]

Several glucocorticoid effects on neutrophils are well documented. Dexamethasone has been reported to enhance granulocyte production by the bone marrow,[19] reduce chemotaxis,[20] suppress $LTB_4$ production,[21] and reduce release of mediators dependent on phospholipase $A_2$.[22] There is evidence that superoxide generation, and release of lactoferrin and lysozyme, is reduced in neutrophils pre-incubated for 20 min with dexamethasone.[23] Lipocortin 1 can inhibit superoxide production.[24] There is, however, a dissenting report that dexamethasone does not inhibit neutrophil chemotaxis, degranulation, $LTB_4$ production, or neutrophil adherence to activated endothelium (*vide infra*).[25]

Glucocorticoid effects on endothelial cells or the vascular bed are poorly defined. As mentioned above,[25] dexamethasone has been reported to be unable to inhibit activated neutrophils adhering to activating endothelium, although it did have a suppressive effect on unstimulated neutrophils adhering to unstimulated endothelium. Dexamethasone has been shown to decrease prostaglandin $I_2$ synthesis by endothelial cells, but not to increase lipocortin I production.[26] Dexamethasone does not reduce arachidonic acid release from endothelial cells,[27] or affect arachidonyl CoA synthetase activity.[28]

There seems then to be a dichotomy between the clinical experience with glucocorticoids and the experimental details obtained with individual tissues *in vivo*. It may be that the effects of glucocorticoids and the lipocortins act 'earlier' or 'upstream' of the neutrophil or endothelial cell. An analogous example might be IL-2, which has marked effects on the endothelium, although none of these effects are direct—rather they are induced by other mediators. It is possible for example that glucocorticoids affect macrophage-type cells, inducing a suppression of inflammatory mediators.

Glucocorticoids are a very effective therapeutic modality. To provide therapy as effective as the glucocorticoids without the side-effects requires an understanding of how they operate. It is clear from this limited study that their effects on the inflammatory process are complex, and are not mediated primarily through adhesion molecules.

### References

1. Hench PS, Boland EW. Potential Reversibility of Rheumatoid Arthritis. *Proc Staff Meeting Mayo Clin* 1949; **24**: 167–168.
2. Conn DL. Update on Systemic Necrotizing Vasculitis. *Mayo Clin Proc* 1989; **64**: 535–543.
3. Lebel MH, Freij BJ, Syrogiannopoulos GA, Chrane DF, Hoyt MJ, Stewart SM, Kennard BD, Olsen KD, McCracken GH. Dexamethasone Therapy for Bacterial Meningitis. Results of Two Double-blind, Placebo-controlled Trials. *N Engl J Med* 1988; **319**: 964–971.
4. Lebel MH, Hoyt MJ, Waagner DC, Rollins NK, McCracken GH. Magnetic Resonance Imaging and Dexamethasone Therapy for Bacterial Meningitis. *Am J Dis Child* 1989; **143**: 301–306.
5. Word BM, Klein JO. Therapy of Bacterial Sepsis and Meningitis in Infants and Children: 1989 poll of directors of programs in pediatric infectious disease. *Pediatr Infect Dis J* 1989; **8**: 635–637.
6. Cummings JJ, D'Eugenio DB, Gross SJ. A Controlled Trial of Dexamethasone in Preterm Infants at High Risk for Bronchopulmonary Dysplasia. *N Engl J Med* 1989; **320**: 1505–1510.
7. Dale DC, Fauci AS, Wolff SM. Alternate Day Prednisone Leukocyte Kinetics and Susceptibility to Infections. *N Engl J Med* 1974; **291**: 1154–1158.
8. Fauci AS, Dale DC, Balow JE. Glucocorticosteroid Therapy: Mechanism of action and clinical considerations. *Ann Int Med* 1976; **84**: 304–315.
9. Bourchier D, Weston PJ. The Effect of Dexamethasone upon Platelets and Neutrophils of Preterm Infants with Chronic Lung Disease. *J Paediatr Child Health* 1991; **27**: 101–104.
10. Barnstable CJ, Bodmer WF, Brown G, Milstein C, Williams AF, Zeigler A. Production of Monoclonal Antibodies to Group A Erythrocytes, HLA and other Human Cell Surface Antigens—new tools for genetic analysis. *Cell* 1978; **14**: 9–20.
11. Wellicome SM, Thornhill MH, Pitzalis C, Thomas DS, Lanchbury JS, Panayi GS, Haskard DO. A Monoclonal Antibody that Detects a Novel Antigen on Endothelial Cells that is Induced by Tumor Necrosis Factor, IL-1, or Lipopolysaccharide. *J Immunol* 1990; **144**: 2558–2565.
12. Keen J, Chang SE, Taylor-Papadimitrou J. Monoclonal Antibodies that Distinguish between Human Cellular and Plasma Fibronectin. *Mol Biol Med* 1984; **2**: 15–27.
13. Forsyth KD, Levinsky RJ. CD15 Antibodies Increase Neutrophil Adherence to Endothelium by an LFA-1 Dependent Mechanism. *Eur J Immunol* 1989; **19**: 1331–1334.
14. Goulding NJ, Godolphin JL, Sharland PR, Peers SH, Sampson M, Maddison PJ, Flower RJ. Anti-inflammatory Lipocortin 1 Production by Peripheral Blood Leucocytes in Response to Hydrocortisone. *Lancet* 1990; 1416–1418.
15. Cirino G, Flower RJ. Human Recombinant Lipocortin 1 Inhibits Prostacyclin Production by Human Umbilical Artery. *Prostaglandins* 1987; **34**: 59–62.
16. Nakano T, Ohara O, Teraoka H, Arita H. Glucocorticoids Suppress Group II Phospholipase $A_2$ Production by Blocking mRNA Synthesis and Post-transcriptional Expression. *J Biol Chem* 1990; **265**: 12745–12748.
17. Mertsolla J, Ramilo O, Mustafa MM, Saez-Llorens X, Hansen EJ, McCracken GH Jr. Release of Endotoxin after Antibiotic Treatment of Gram Negative Bacterial Meningitis. *Pediatr Infect Dis J* 1989; **8**: 904–906.

18. Knudsen PJ, Dinarello CA, Strom TB. Glucocorticoids Inhibit Transcriptional and Post-transcriptional Expression of Interleukin 1 in U937. *J Immunol* 1987; **139**: 4129–4134.
19. Shezen E, Shirman M, Goldman R. Opposing Effects of Dexamethasone on the Clonal Growth of Granulocyte and Macrophage Progenitor Cells and on the Phagocytic Capability of Mononuclear Phagocytes at Different Stages of Differentiation. *J Cell Physiol* 1985; **124**: 545–548.
20. Ward PA. The Chemosuppression of Chemotaxis. *J Exp Med* 1966; **124**: 209–226.
21. Schreiber AD, Parson J, McDermott P, Cooper RA. Effect of Corticosteroids on the Human Monocyte IgG and Complement Receptors. *J Clin Invest* 1975; **56**: 1189–1197.
22. Blowers LE, Jayson MI, Jasani MK. Effect of Dexamethasone on Polypeptide Synthesized in Polymorphonuclear Leukocytes. *FEBS Lett* 1985; **181**: 362–366.
23. Coates TD, Wolach B, Tzeng DY, Higgins C, Baehner RL, Boxer LA. The Mechanism of Action of the Anti-inflammatory Agents Dexamethasone and Auranofin in Human Polymorphonuclear Leukocytes. *Blood* 1983; **62**: 1070–1077.
24. Stevens TRJ, Drasdo AL, Peers SH, Hall ND, Flower RJ. Stimulus Specific Inhibition of Human Neutrophil $H_2O_2$ Production by Human Recombinant Lipocortin 1. *Br J Pharmacol* 1988; **93**: 139–143.
25. Schleimer RP, Freeland HS, Peters SP, Brown KE, Derse CP. An Assessment of the Effects of Glucocorticoids on Degranulation, Chemotaxis, Binding to Vascular Endothelium and Formation of Leukotriene $B_4$ by Purified Human Neutrophils. *J Pharmacol Exp Ther* 1989; **250**: 598–605.
26. Hullin F, Raynal P, Ragab-Thomas JM, Fauvel J, Chap H. Effect of Dexamethasone on Prostaglandin Synthesis and on Lipocortin Status in Human Endothelial Cells. Inhibition of Prostaglandin $I_2$ Synthesis occurring without Alteration of Arachidonic Acid Liberation and of Lipocortin Synthesis. *J Biol Chem* 1989; **264**: 3506–3513.
27. Medow MS, Intrieri L, Moatter T, Gerritsen ME. Dexamethasone Effects on Microvascular Endothelial Cell Lipid Composition. *Am J Physiol* 1989; **257**: C512–519.
28. Gerritsen ME, Perry CA. Regulation of Eicosanoid Synthesis in Microvascular Endothelium: Glucocorticoids do not affect arachidonyl CoA synthetase activity. *Biochim Biophys Acta* 1990; **1045**: 174–179.

ACKNOWLEDGEMENT. This work was supported by the Princess Margaret Rheumatoid Arthritis Research Fund.

**Received 8 October 1991; accepted in revised form 7 January 1992**
